# Modern Surgical Site Infection Prevention: Evidence, Gaps, and Future Directions

**DOI:** 10.7759/cureus.94764

**Published:** 2025-10-17

**Authors:** Raghunath Prabhu, Mohamed S Mohamed, Tariq Alhammali, Raouf Ghareb, Suhas Doddamane Prasanna, Momen Abdelglil, Ali Soffar, Ahmed Elkohail, Mahmoud Teama, Nervana Khalil, Ahmed Elhantiry

**Affiliations:** 1 Colorectal Surgery, Leicester Royal Infirmary, University Hospitals of Leicester NHS Trust, Leicester, GBR; 2 General Surgery, Belfast Health and Social Care Trust, Belfast, GBR; 3 Colorectal Surgery, University Hospitals of Leicester NHS Trust, Leicester, GBR; 4 General and Colorectal Surgery, Royal Free NHS Trust, Barnet Hospital, Barnet, GBR; 5 Internal Medicine, Leicester General Hospital, University Hospitals of Leicester NHS Trust, Leicester, GBR; 6 Pediatric Surgery, Mansoura University Children Hospital, Mansoura, EGY; 7 Trauma and Orthopaedics, Princess Royal University Hospital, Kings College NHS Foundation Trust, London, GBR; 8 General Medicine, King's College Hospital, London, GBR; 9 Anesthesia and Critical Care, The Memorial Souad Kafafi University Hospital, 6th of October City, EGY

**Keywords:** antimicrobial stewardship, artificial intelligence & machine learning, multidrug resistance, postoperative remote surveillance, ssi, surgical site infection

## Abstract

Surgical site infections (SSIs) remain among the most consequential and costly complications in surgery. Their persistence reflects the expanding threat of multidrug-resistant (MDR) organisms and the practical limits of even increasingly data-driven, systems-level prevention programs. Contemporary prevention is multimodal across the perioperative pathway. Preoperatively, patient optimization is central, and foundational measures include alcohol-based skin antisepsis, nutritional support, smoking cessation, and strict glycemic control. Intraoperatively, the emphasis shifts to technical precision and physiologic stability, including gentle tissue handling, maintenance of normothermia, appropriate oxygenation, and disciplined operating-room traffic. In parallel, material science and precision medicine are reshaping the armamentarium: antimicrobial sutures and surface coatings, host-directed immunomodulation, and personalized, risk-adapted strategies offer targeted protection. This narrative review synthesizes current evidence across these domains. We aim to clarify which measures carry the strongest support, identify persistent gaps and implementation challenges, and outline priorities for future research and practice.

## Introduction and background

Surgical site infection (SSI) remains one of the most consequential healthcare-associated complications, typically affecting about 2-5% of surgical patients worldwide and varying by region and procedure complexity [1,2]. Beyond the significant burden of its incidence, the economic and human toll is substantial; classic analyses estimate an average 9.7-day prolongation of hospitalization and an incremental cost of roughly $20,842 per admission, figures that continue to anchor contemporary burden estimates and quality-improvement targets [3,4]. The landscape is further complicated by the rising prevalence of multidrug-resistant (MDR) organisms in surgical care, which is linked to higher morbidity and mortality and increasingly undermines the effectiveness of standard antibiotic prophylaxis, particularly when guideline adherence is suboptimal or local resistance patterns shift [5-7]. 

Minimally invasive and robot-assisted procedures, which limit tissue injury, are associated with a lower risk of infection. Smaller incisions, less blood loss, and shorter hospitalizations are some of the benefits of these techniques, collectively reducing the likelihood of SSIs [5,6]. Embedding analytics and active surveillance in SSI programs enables the data-driven identification of high-risk patients and targeted prevention efforts. Multidisciplinary teams can leverage large datasets to uncover patterns, trends, and risk factors. Predictive models can flag patients at elevated risk, supporting preemptive measures and tailored prophylaxis [7]. This review synthesizes current evidence-based prevention strategies, appraises emerging technologies and novel approaches, and outlines future research priorities and practical implementation pathways to reduce SSI across diverse settings.

## Review

Methods

We conducted a narrative, thematically organized review using a predefined concept grid (pre-, intra-, postoperative SSI prevention; MDR/antimicrobial strategies; digital/AI tools), accompanied by iteratively refined keywords and Boolean operator "surgical site infection" AND (prevention OR prophylaxis) AND (decolonization OR "negative-pressure wound therapy" OR MRSA). Sources included PubMed/MEDLINE, Embase, and Cochrane, guideline repositories (e.g., CDC/WHO), targeted society statements, and hand-searching reference lists of included studies to minimize missed evidence. The search was limited to English-language items, and we hence acknowledge possible language-related bias.

Literature (guidelines, consensus statements, high-quality papers) was appraised for authority, methodological transparency, recency, and triangulation with peer-reviewed data; preprints were used cautiously. No protocol was prospectively registered (e.g., PROSPERO) given the narrative scope; we nevertheless report search domains, selection logic, and synthesis approach for transparency. Inter-rater reliability was not formally quantified; disagreements were resolved by discussion.

Evidence was synthesized narratively by domain, with preference for higher-level evidence (systematic reviews, randomized controlled trials (RCTs), multicenter cohorts) and externally validated/consensus-backed work; single case reports were excluded unless uniquely illustrative. We did not perform a formal risk-of-bias assessment but considered study design, confounding control, sample size, and consistency across sources when interpreting themes.

Current evidence-based prevention strategies

Patient Screening and Risk Stratification

In contemporary programs, preoperative risk work starts with pathogen-focused screening layered onto global risk calculators. For Staphylococcus aureus (S. aureus), targeted preoperative screening with nasal polymerase chain reaction (PCR) or culture, followed by a short course of intranasal mupirocin plus whole-body chlorhexidine gluconate (CHG) bathing, remains a pragmatic, evidence-supported bundle that can lower S. aureus SSI, particularly in orthopedic and implant surgery when implemented with high fidelity and timely dosing (typically five days) [8]. 

For carriers of MDR Gram-negative bacteria (MDR-GNB), the 2023 European Society of Clinical Microbiology and Infectious Diseases (ESCMID)/the European Committee on Infection Control (EUCIC) guidelines move beyond blanket approaches and endorse selective rectal screening with targeted perioperative prophylaxis in defined scenarios (e.g., fluoroquinolone-resistant Enterobacterales before transrectal prostate biopsy; extended-spectrum cephalosporin-resistant Enterobacterales in colorectal surgery and solid-organ transplantation), while suggesting pretransplant screening for carbapenem-resistant Enterobacterales (CRE) and carbapenem-resistant Acinetobacter baumannii (CRAB) [9]. 

Randomized data show that screening surgical candidates for S. aureus and decolonizing carriers with intranasal mupirocin plus chlorhexidine bathing halves postoperative S. aureus infections in carriers, notably in orthopedic/cardiothoracic cohorts; postdischarge decolonization similarly reduces subsequent methicillin-resistant Staphylococcus aureus (MRSA) infections, reinforcing preoperative, pathogen-focused bundles [10,11].

Modern risk stratification should extend beyond pathogen carriage to modifiable host factors and calibrated prediction. Embedding dynamic tools (e.g., American College of Surgeons National Surgical Quality Improvement Program risk estimates) into preadmission workflows helps surface high-risk patients early and target bundles (decolonization, glucose control, nutrition, anemia management) where they will yield the greatest absolute risk reduction [12,13].

Antimicrobial Prophylaxis Strategies

Classic perioperative prophylaxis still may prevent most SSIs when executed with precision, but the MDR era demands sharper adjustment of prophylaxis. The first dose should be administered within 60 minutes of incision (up to 120 minutes for vancomycin or fluoroquinolones), followed by redosing intraoperatively when procedures outlast two drug half-lives or when blood loss is substantial, and avoiding routine postoperative continuation beyond the operating room (OR) or post-anesthesia care unit (PACU) except in narrowly defined circumstances [14,15]. 

In patients colonized with MRSA, major guidelines and recent analyses converge: reserve vancomycin prophylaxis for patients with documented MRSA colonization (and consider for hip/knee arthroplasty where screening is impractical), rather than using it broadly, both to protect cefazolin’s superior anti-methicillin-susceptible S. aureus performance and to minimize nephrotoxicity [15,16]. For institutions grappling with high local resistance, stewardship teams should (i) codify decision trees that (i) default to cefazolin-based regimens for most clean procedures, (ii) add vancomycin only for verified MRSA carriers or selected implant cases, and (iii) apply targeted MDR-GNB prophylaxis only where guideline-supported and microbiology-confirmed, with explicit plans to stop at wound closure [15,17].

Recent evidence reinforces the importance of optimal timing and intraoperative redosing to maintain prophylactic coverage during prolonged procedures. A 2024 systematic review/meta-analysis found lower SSI risk with redosing versus no redosing (OR: 0.65, 95% CI: 0.45-0.94), with the clearest benefit in studies without postoperative antibiotic continuation and in operations lasting ≥4 hours [18]. Observational surgical cohorts similarly report reduced SSI with redosing in prolonged cases, reinforcing practice across procedures [19]. Accordingly, guideline-based protocols recommend redosing at two half-lives or with major blood loss (≥1500 mL), with representative intervals of cefazolin every four hours and clindamycin every six hours, and stopping prophylaxis at wound closure [15]. Dosing should also be weight-aware; for patients ≥120 kg, many programs use cefazolin 3 g to achieve adequate tissue levels throughout the operation [20].

Preoperative and Intraoperative Measurements

Skin antisepsis with an alcohol-based agent is important, as many guidelines favor CHG-alcohol over povidone-iodine (PVI) on the strength of earlier meta-analyses, though several newer randomized and comparative studies show non-inferiority of PVI-alcohol to CHG-alcohol in cardiac and other major surgeries, so the practical message is to use an alcohol-based formulation, applied meticulously and allowed to dry, rather than to fixate on the molecule when high-quality products and technique are ensured [21,22]. Identified protein-energy deficits should be corrected with individualized, high-protein regimens, and dietitian-led interventions should be integrated to reduce downstream postoperative wound problems[23,24].* *

Structured programs producing ≥4 weeks of abstinence before surgery reduce wound complications and overall adverse events and should be coupled with nicotine replacement and behavioral support embedded in preop clinics [25,26]. Perioperative glycemic control should be embedded in the preoperative checklist for patients with diabetes and stress hyperglycemia. A target range of 80-180 mg/dL is preferred over “tight” control (<110 mg/dL), which has not reliably decreased SSI rates and heightens hypoglycemia risk. Monitoring and insulin management should be standardized in accordance with American Diabetes Association (ADA) society recommendations [17,27].

Gentle tissue handling, hemostasis, and avoidance of unnecessary foreign material remain important things. Two physiologic pillars deserve special attention: maintaining normothermia and ensuring adequate oxygenation. Landmark randomized trials demonstrate that maintaining intraoperative normothermia reduces wound infections and expedites recovery; warming should begin before incision (prewarming), continue intraoperatively, and be paired with warmed IV fluids and active thermal monitoring as the default, especially for long colorectal and major cases [28,29]. 

**Figure 1:**
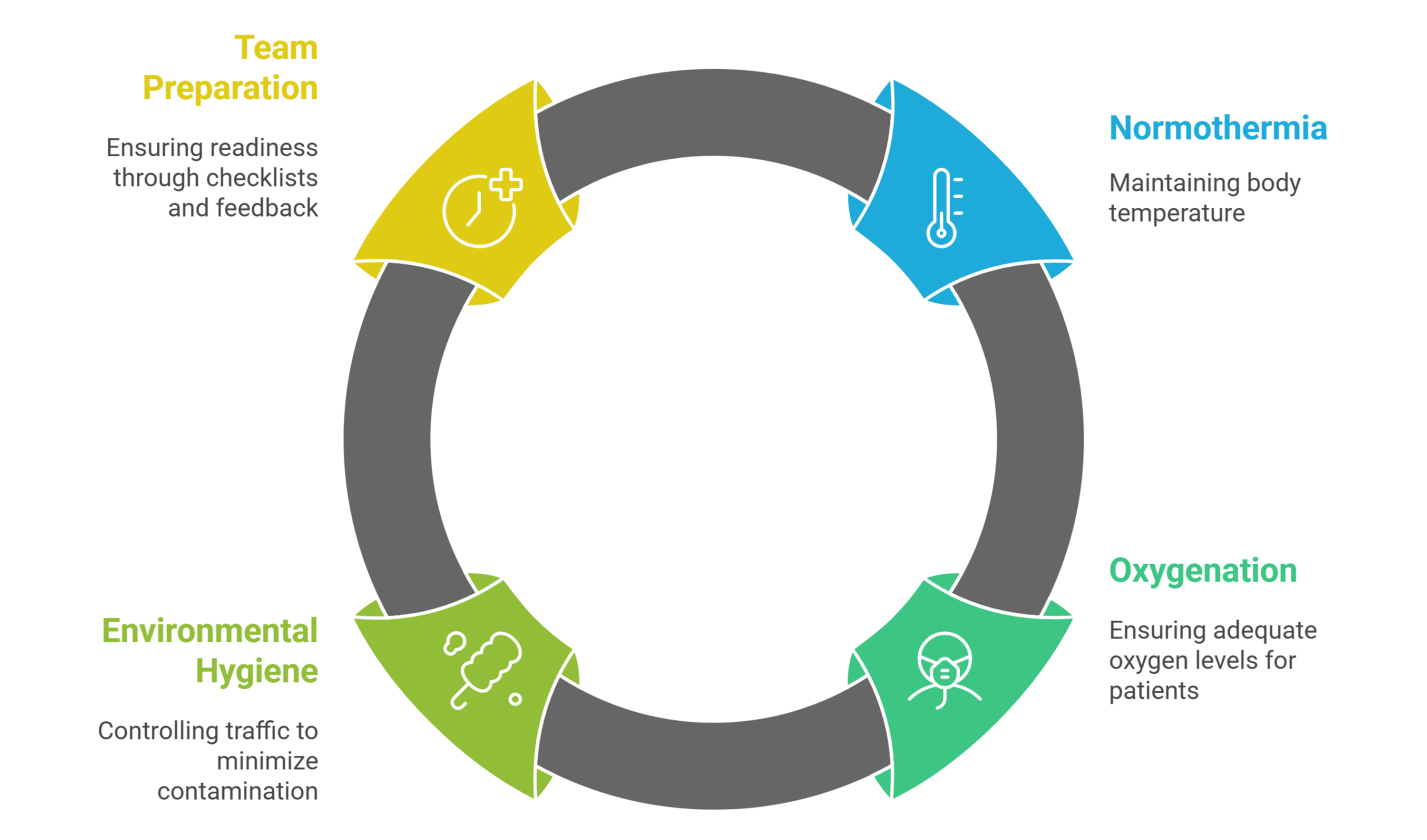
A summary of intraoperative measures for reducing SSI Source: [27-31] Figure credit: Momen Abdelglil SSI: surgical site infection

Operating-room environmental hygiene complements aseptic technique, so traffic control, such as reducing door openings and crowding, has biologic plausibility and emerging quantitative support Individual-patient meta-analysis suggests each additional door opening per hour adds a small but cumulative increase in SSI risk, and multiple studies link door openings to higher airborne bioburden; practical measures like access-restriction signage and role-based zoning can cut unnecessary entries [30,31]. Team preparation, scrub technique, sterile gowning and gloving, standardized field setup, and surgical safety checklists should be audited as essential practices, with real-time coaching and feedback loops from infection-prevention and OR nursing leadership (Figure 1) [17].

Postoperative Care

Postoperative wound care should be protocolized yet individualized, for standard-risk incisions simple sterile dressings left undisturbed for 24-48 hours with patient education on hygiene and symptom recognition usually suffice, in high-risk incisions-for example obesity, contaminated fields, long procedures, or extensive soft-tissue dissection-closed-incision negative-pressure wound therapy (ciNPWT) can reduce surgical site infection and dehiscence in several populations including obstetric, spine, and abdominal cohorts though results are not uniform across all leg or vascular incisions, therefore criteria-based use anchored to factors such as BMI, wound class, perfusion status, and implant presence together with shared decision-making is prudent [32-34]. Early mobilization improves global outcomes and likely lowers infection risk by enhancing ventilation, perfusion, and glycemic control. Practical targets (e.g., ambulation within 24-36 hours when feasible) and removal of barriers (e.g., lines, strict bedrest orders) should be embedded in order sets [35,36]. Also, surveillance-friendly wound assessments (including photo-enabled remote monitoring where infrastructure allows) facilitate earlier detection and intervention without increasing clinic burden [37,38].

Prophylactic antibiotics should not be continued beyond wound closure, except in clearly delineated contexts (e.g., select cardiothoracic or transplant procedures) specified by institutional policy. Extending “just-in-case” courses has not demonstrated SSI reduction and instead promotes antimicrobial resistance and Clostridioides difficile infection [14,15].

Impact of artificial intelligence (AI) integration on surgical site infection prevention

AI is shifting SSI prevention from checklist-based methods to proactive, data-driven strategies that predict risk, ensure standardized care, and enhance early detection after discharge. Preoperatively, machine learning models using routine EHR data often show strong accuracy (AUC ≥0.80), supporting targeted measures such as glucose control and MRSA/MDR screening. Reviews indicate that validated, Transparent Reporting of a multivariable prediction model for Individual Prognosis Or Diagnosis (TRIPOD) compliant tree ensembles and deep learning models, especially those using text and temporal features, outperform conventional logistic regression and demonstrate reliable calibration. [39-41]. Digital order sets and real-time decision support can track critical steps such as correct prophylactic drug, dose, timing, scheduled redosing, and normothermia checks while prompting teams if an omission is likely [42,43].

A second frontier is smart dressings and wearables that convert wound-bed biology into discrete data streams readable by algorithms. Flexible printed sensors measuring temperature, pH, moisture, oxygen tension, and volatile amines can infer shifts toward infection before clinical erythema or drainage, while AI filters suppress noise from movement, ambient conditions, and dressing changes. Early human and translational studies describe on-dressing analytics that alert patients and care teams, with reviews highlighting paths to closed-loop systems that could trigger on-demand interventions (e.g., local heat, electrical stimulation, or topical antimicrobials) once thresholds are crossed [44,45].

Direct, multicenter evidence that toggling “AI on” versus “AI off” reduces SSI incidence remains limited but is steadily emerging. At present, the most persuasive signals lie in intermediate outcomes along the causal pathway: enhanced adherence to prophylaxis, more precise risk stratification, earlier detection, and faster clinical response. In emergency and gastrointestinal surgery cohorts, trials and implementation pilots of digital wound-care pathways have shortened time to diagnosis and optimized resource use without compromising safety, a plausible mechanism for reducing superficial SSI where treatment delay is pivotal [37,46].

Digital twins are an emerging direction in AI-assisted wound care: dynamic virtual replicas of wounds that update with real-time sensor data and clinical observations to simulate healing trajectories, assess infection risk, and test “what-if” treatment scenarios before bedside implementation [39,47]. Early implementations integrate multimodal sensors such as pH, temperature, and inflammatory biomarkers with AI pipelines to generate personalized healing predictions and support timely intervention, while scenario-maker modules in prototype platforms allow clinicians to explore alternative therapies virtually and optimize care pathways, and although rigorous external validation in surgical settings remains limited the technology trajectory suggests a feasible path to privacy-preserving, data-driven wound management at scale [45,48].

AI is increasingly integrated with antimicrobial stewardship programs (ASPs) to improve prophylaxis quality and curb resistance. Machine-learning models that combine patient factors with local resistance data can guide antimicrobial selection, with reported AUCs ranging from ~0.64 to 0.992 across stewardship tasks. EHR-embedded decision-support tools can monitor compliance with surgical antibiotic prophylaxis protocols, automatically flag deviations from guidelines, and suggest evidence-based corrections-supporting reductions in inappropriate prescribing while maintaining infection-prevention aims. Further multicenter, SSI-focused evaluations are needed to confirm clinical impact across diverse surgical settings [49,50].

**Figure 2:**
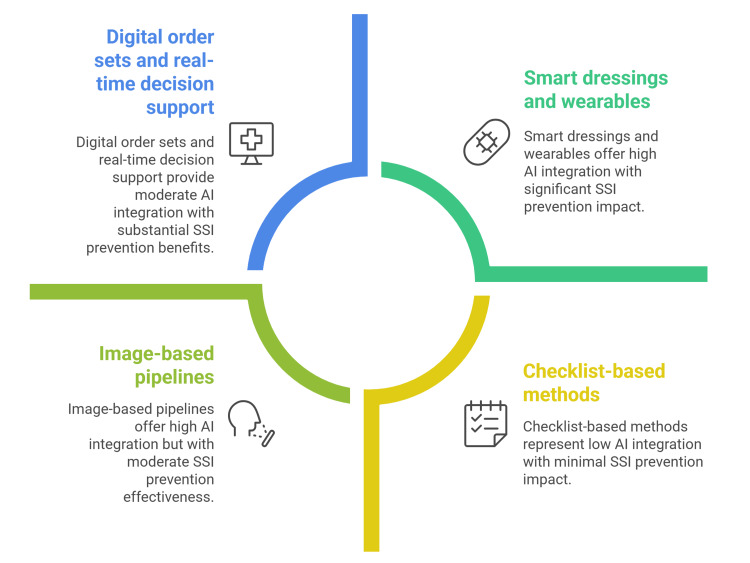
Impact of integrating AI on SSI care Source: [41,53] Figure credit: Momen Abdelglil AI: artificial intelligence; SSI: surgical site infection

Despite promising results, the generalizability of AI-based SSI prediction remains a major hurdle. Federated learning (FL) enables multi-institution collaboration without exchanging raw data, and recent implementations show that FL can achieve performance comparable to centralized training while preserving privacy and institutional data control. Reports of FL using standard algorithms such as federated averaging (FedAvg) document strong discrimination (often AUC >0.90) on clinical prediction tasks across sites. Yet, external validation is still uncommon, and cross-site performance can degrade without deliberate strategies to handle data heterogeneity, underscoring the need for collaborative, multicenter validation frameworks to establish clinical utility across diverse populations (Figure 2) [39,51-53].

Novel antimicrobial strategies

Novel antimicrobial strategies for SSI prevention increasingly address multidrug-resistant organisms through innovative approaches and combinations. One cornerstone involves antimicrobial-impregnated sutures, such as those coated with triclosan or chlorhexidine, which actively inhibit bacterial colonization and biofilm formation around foreign materials, thereby reducing SSI rates in various surgical settings. These antiseptic sutures have shown effectiveness against common pathogens, including S. aureus and Escherichia coli, though resistance concerns prompt ongoing refinement of their formulation [54,55].

Combination antibiotic therapies represent another critical strategy, especially for high-risk or MDR infections. Utilizing two or more agents with different mechanisms can enhance antimicrobial coverage, provide synergistic effects, and combat pathogens like carbapenem-resistant Enterobacteriaceae more effectively than monotherapy, although this approach should be judiciously reserved for severe or life-threatening scenarios to avoid contributing to resistance. Empirical usage is often limited to the early phases of critical illness, with definitive single-agent treatments preferred once pathogens are identified [56,57]

Immunomodulatory approaches have garnered attention for their ability to harness and accelerate the innate immune response at the surgical site. Agents such as formyl-Methionine-Lysine-Proline (fMLP) and other chemokines have demonstrated the ability to boost inflammation, enhance bacterial clearance, and improve healing in experimental SSI models, proving to be as effective as systemic antibiotics without promoting resistance or adverse healing outcomes. Such host-directed therapies may offer broad-spectrum, pathogen-agnostic protection when combined with standard prophylactic measures [58].

Precision medicine strategies are now being explored for SSI management, aiming to tailor antimicrobial prevention to each patient’s risk profile, microbiota, and genetic background. By leveraging advances in pathogen detection, antibiotic stewardship, and genomic analysis, clinicians can select individualized prophylactic regimens, optimize dosing, and reduce unnecessary exposure, thereby maximizing efficacy and minimizing resistance development. As these approaches mature, SSI prevention is being transformed into a more targeted, data-driven discipline poised to overcome longstanding challenges associated with multidrug resistance [59-61].

Implementation challenges, considerations, and future directions

Implementation of SSI prevention strategies faces significant barriers that compromise their effectiveness in clinical practice. A key challenge is insufficient compliance with infection control bundles; even evidence-based protocols often fail to reduce SSI rates when adherence to individual measures, such as temperature control, wound protection, and closure techniques, is suboptimal. The success of prevention bundles relies heavily on high compliance, which is difficult to maintain in busy or understaffed surgical environments [62]. Surveillance and monitoring systems are crucial for identifying and reducing SSIs. Yet, many institutions, especially in low- and middle-income countries, lack standardized reporting, robust surveillance infrastructure, and clear definitions for SSIs. These limitations result in significant underreporting and reduced accountability, hindering efforts to target improvement interventions effectively [63,64].

Future directions in SSI prevention will increasingly emphasize the integration of advanced technologies with traditional clinical practices to enhance patient outcomes and combat multidrug resistance. One of the most promising developments is the use of AI and machine learning to predict individual SSI risk in real time. These technologies can analyze complex clinical and procedural data to provide personalized recommendations for preoperative optimization, intraoperative management, and postoperative care, thereby facilitating targeted interventions that improve efficiency and efficacy in infection prevention [53,61].

The evolution of antimicrobial coatings and implantable devices represents another critical frontier. Innovations in polymer science and nanotechnology are enabling the controlled release of antibacterial agents from implants and sutures, which can prevent bacterial colonization and biofilm formation on surgical sites. Future research aims to optimize these materials for biocompatibility, durability, and broad-spectrum antimicrobial activity while minimizing resistance development. These smart biomaterials promise to transform SSI prevention by providing long-lasting, localized defense against pathogens [61,65,66].

Implementation should explicitly account for resource constraints and baseline risk, which are substantially higher in many low- and middle-income settings. The GlobalSurg multicentre cohort reported markedly greater SSI incidence in low-HDI countries versus high-HDI settings, underscoring the need to prioritize low-cost, high-yield bundles, reliable supply of alcohol-based antiseptics, and basic surveillance capacity [67].

Using a smartphone app for follow-up care after surgery can help doctors find wound infections more effectively and possibly earlier than with routine office visits. In this system, patients send photos of their surgical wound and report any symptoms through an app that is monitored by their doctor. Studies show that patients are satisfied with this method, finding it comparable to in-person visits and even reporting a better quality of recovery. For this system to work well, healthcare providers need a clear plan to review patient submissions and should use simple, affordable apps so more people can access the service [68,69].

**Figure 3:**
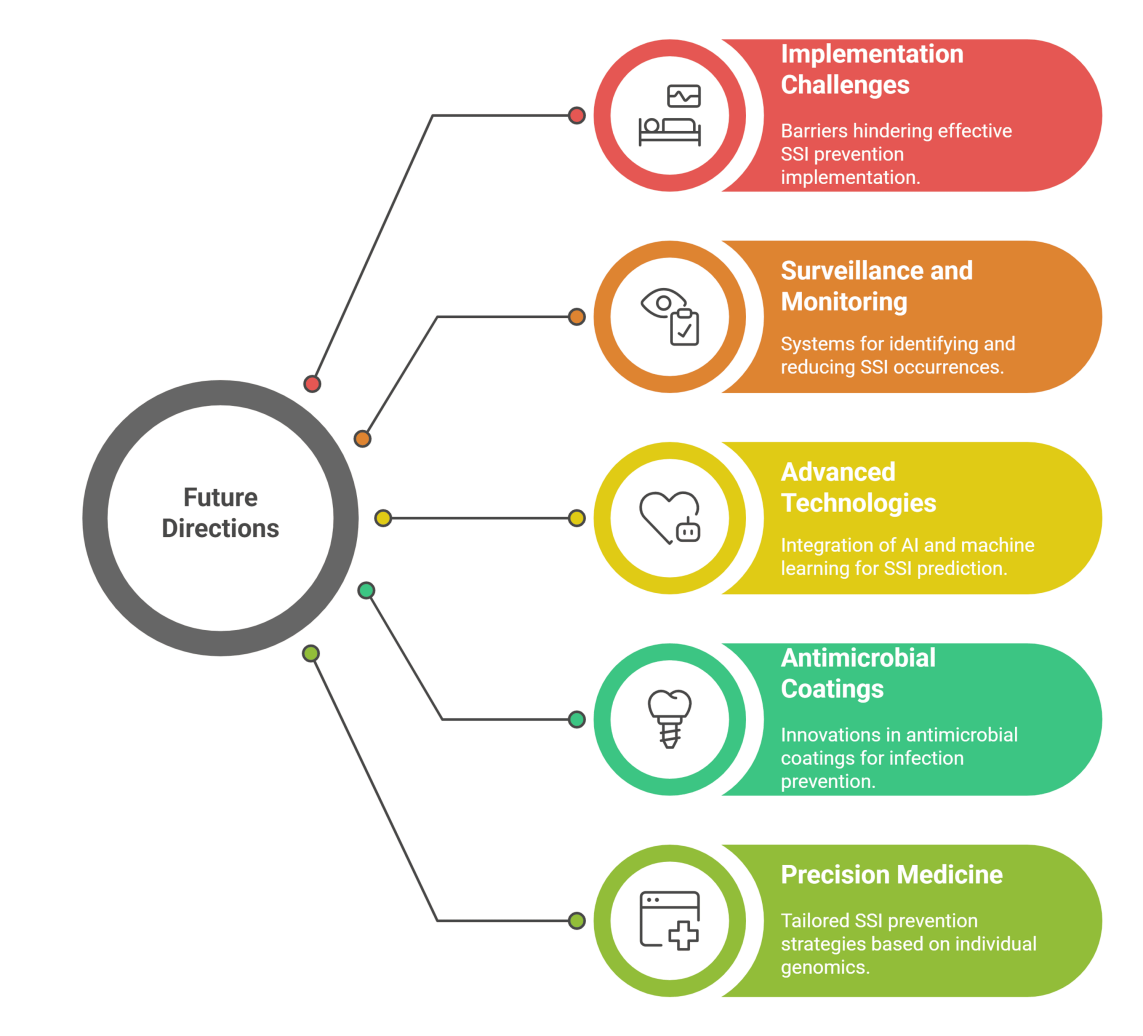
Implementation challenges, considerations, and future directions for SSI management Source: [62-66] Figure credit: Momen Abdelglil AI: artificial intelligence; SSI: surgical site infection

Precision medicine approaches will further refine SSI prevention by tailoring prophylactic strategies based on individual patient genomics, microbiome profiles, and immune responses. This personalized approach allows for more accurate identification of patients at high risk for MDR infections and enables customized antimicrobial regimens that maximize preventive benefits while reducing unnecessary antibiotic exposure. Integrating pharmacogenomics and microbial genomics into surgical care pathways will enhance precision and pave the way for innovative targeted therapies (Figure 3) [59,60]. This review underscores prioritizing high-certainty, cross-setting measures and rigorous implementation over product substitution. Future research should include multicenter, procedure-specific trials, standardized outcomes, cost-effectiveness and equity analyses, external validation of predictive tools, and pragmatic implementation studies - particularly in low-resource settings - linking process improvements and digital surveillance to reductions in surgical site infection incidence.

## Conclusions

Modern SSI prevention achieves optimal outcomes when foundational practices are executed flawlessly alongside innovative, context-sensitive strategies. Essential measures include targeted pathogen screening and decolonization, precise guideline-based antimicrobial prophylaxis with timely administration and cessation at wound closure, alcohol-based skin antisepsis, optimized patient factors such as nutrition and glycemic control, meticulous surgical technique, maintenance of intraoperative normothermia, disciplined operating room traffic control, and structured postoperative care. These core practices form the basis upon which technological advances such as remote photo-enabled wound monitoring and AI-driven early-warning systems can be effectively layered to accelerate infection detection and intervention. 

The principal challenge to advancing SSI prevention is inconsistent implementation rather than a lack of evidence or novel ideas. Priority efforts should focus on adapting strategies to local microbial epidemiology and antimicrobial stewardship frameworks, selectively screening for MDR-GNB with tailored prophylaxis in supported contexts, and establishing robust measurement and feedback systems that directly link compliance with clinical outcomes. In settings equipped for digital innovations, piloting AI-based risk prediction models and sensor-augmented wound surveillance pathways is encouraged, with attention to feasibility, equity, cost, and patient safety. Persistent gaps include optimal strategies for MDR-GNB screening, the generalizability and cost-effectiveness of AI tools, variable applicability in low-resource settings, and the underexplored roles of patient engagement and long-term outcome tracking.
